# Metabolic engineering of *Aspergillus niger* via ribonucleoprotein-based CRISPR–Cas9 system for succinic acid production from renewable biomass

**DOI:** 10.1186/s13068-020-01850-5

**Published:** 2020-12-14

**Authors:** Lei Yang, Mikkel Møller Henriksen, Rasmus Syrach Hansen, Mette Lübeck, Jesper Vang, Julie Egelund Andersen, Signe Bille, Peter Stephensen Lübeck

**Affiliations:** 1 Section for Sustainable Biotechnology, Department of Chemistry and Bioscience, Aalborg University Copenhagen, A. C. Meyers Vænge 15, 2450 Copenhagen SV, Denmark; 2 Section of Microbiology, Department of Biology, University of Copenhagen, Universitetsparken 15, 2100 Copenhagen, Denmark; 3 Section of Cell and Neurobiology, Department of Biology, University of Copenhagen, Universitetsparken 15, 2100 Copenhagen, Denmark; 4 Disease Data Intelligence, Department of Health Technology Bioinformatics, Technical University of Denmark, Bldg. 208, 2800 KemitorvetKgs. Lyngby, Denmark

**Keywords:** *Aspergillus niger*, Metabolic engineering, Succinic acid production, CRISPR–Cas9 system

## Abstract

**Background:**

Succinic acid has great potential to be a new bio-based building block for deriving a number of value-added chemicals in industry. Bio-based succinic acid production from renewable biomass can provide a feasible approach to partially alleviate the dependence of global manufacturing on petroleum refinery. To improve the economics of biological processes, we attempted to explore possible solutions with a fungal cell platform. In this study, *Aspergillus niger*, a well-known industrial production organism for bio-based organic acids, was exploited for its potential for succinic acid production.

**Results:**

With a ribonucleoprotein (RNP)-based CRISPR–Cas9 system, consecutive genetic manipulations were realized in engineering of the citric acid-producing strain *A. niger* ATCC 1015. Two genes involved in production of two byproducts, gluconic acid and oxalic acid, were disrupted. In addition, an efficient C_4_-dicarboxylate transporter and a soluble NADH-dependent fumarate reductase were overexpressed. The resulting strain SAP-3 produced 17 g/L succinic acid while there was no succinic acid detected at a measurable level in the wild-type strain using a synthetic substrate. Furthermore, two cultivation parameters, temperature and pH, were investigated for their effects on succinic acid production. The highest amount of succinic acid was obtained at 35 °C after 3 days, and low culture pH had inhibitory effects on succinic acid production. Two types of renewable biomass were explored as substrates for succinic acid production. After 6 days, the SAP-3 strain was capable of producing 23 g/L and 9 g/L succinic acid from sugar beet molasses and wheat straw hydrolysate, respectively.

**Conclusions:**

In this study, we have successfully applied the RNP-based CRISPR–Cas9 system in genetic engineering of *A. niger* and significantly improved the succinic acid production in the engineered strain. The studies on cultivation parameters revealed the impacts of pH and temperature on succinic acid production and the future challenges in strain development. The feasibility of using renewable biomass for succinic acid production by *A. niger* has been demonstrated with molasses and wheat straw hydrolysate.

## Background

With increasing concerns on climate change and its adverse impacts on the natural environment as well as human society, development of biotechnology has been focusing on providing sustainable and environment-friendly solutions for global manufacturers of commodity chemicals, which heavily rely on oil refinery and give high greenhouse gas emission and environmental pollution. For the past decades, bio-based succinic acid production from renewable biomass has been one of the research hotspots in sustainable biotechnology due to the great potentials of succinic acid to be a building block for deriving varieties of commodity and specialty chemicals [1]. Furthermore, bio-based succinic acid production is also considered as a promising paradigm demonstrating the feasibility of using sustainable biological processes to replace petrol-based processes in the chemical industry [2]. With great efforts in process development and strain improvement, the price of succinic acid produced from biological processes using microbial strains (*Actinobacillus succinogenes*, *Escherichia coli*, and *Saccharomyces cerevisiae*) has been lowered to a competitive level compared with that produced via petrol-based processes [3, 4]. However, if the further application of succinic acid aims at becoming a new bio-based building block in chemical industry to produce commodity chemicals, such as 1,4-butanediol and biodegradable polymers, the current price is still too expensive, which demands further improvement on the economics of biological processes. Production strains are of great importance to process development for bio-succinic acid production. The performance of microbial strains does not only determine the process productivity, but also influences the choice of feedstock and designing of downstream processes involving product purification. Although a number of biological processes have successfully been commercialized with bacterial and yeast strains, some limitations have also been exposed in these strains, which restrict the strain improvement on several key limiting factors in succinic acid production. Among limiting factors are utilization of cheap substrates, yield and productivities of succinic acid, and tolerance of stress conditions, including pH and other potential inhibitors [5, 6]. In order to break through the technical bottlenecks, more research attention has been directed on exploitation of new microbial platforms for bio-succinic acid production [7, 8].

**Fig. 1:**
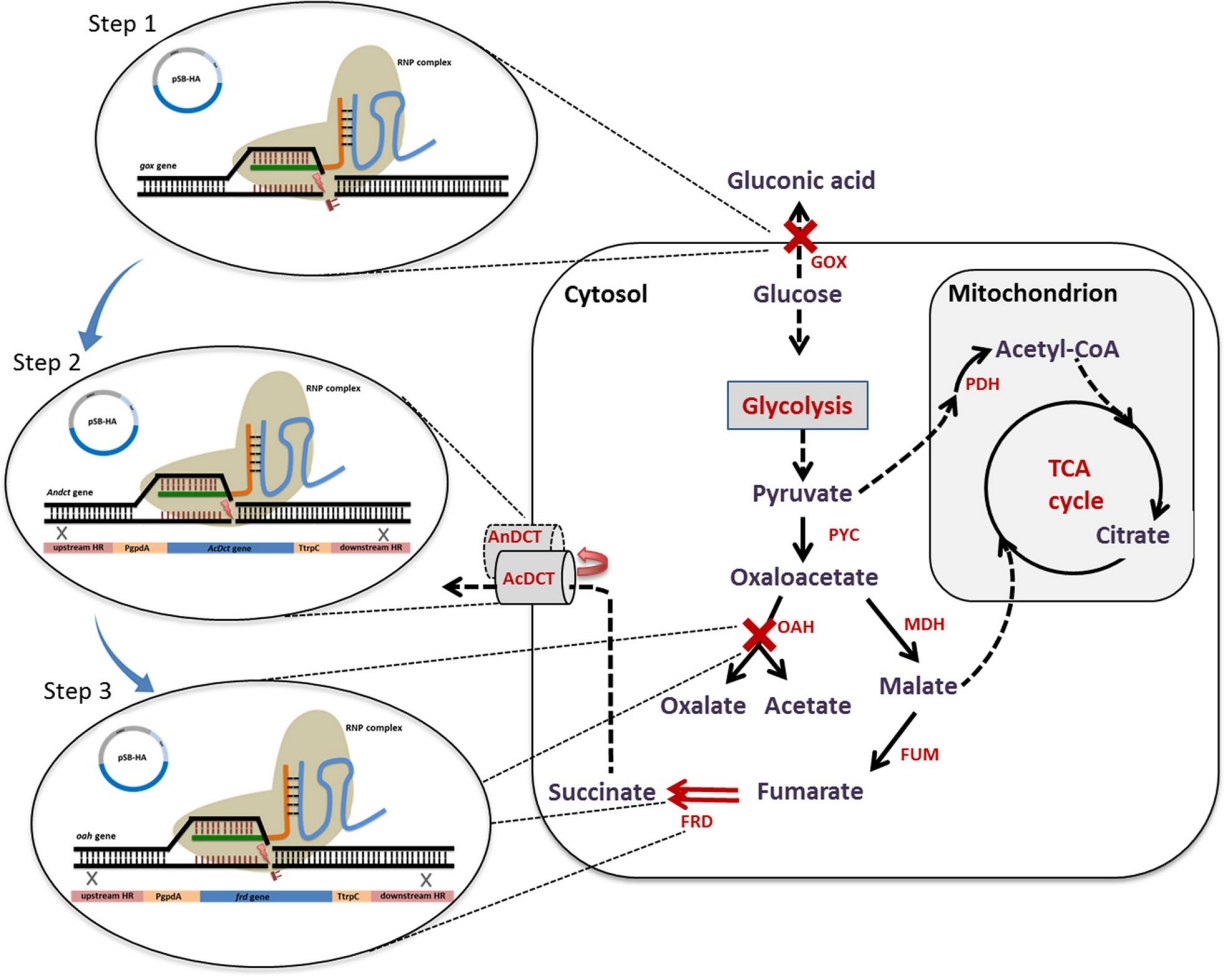
Proposed metabolic pathway for succinic acid production by *A. niger* and strategy for strain engineering (the SAP-3 strain). PYC, pyruvate carboxylase; MDH, malate dehydrogenase; FUM, fumarase; FRD, fumarate reductase; AnDCT, C4-dicarboxylate transporter from *A. niger*; AcDCT, C4-dicarboxylate transporter from *A. carbonarius*; PDH, pyruvate dehydrogenase; GOX, glucose oxidase; OAH, oxaloacetate hydrolase; TCA cycle, tricarboxylic acid cycle

In the history of microbial production of organic acids, *Aspergillus niger* has been well known as a successful industrial workhorse for bio-based production of citric acid. It possesses a number of virtues that greatly facilitate development of biological processes for organic acid production, e.g., efficient secretion of organic acids, excellent capabilities of utilizing varieties of cheap feedstock, high tolerance to stress conditions, e.g., low pH and heavy metals [9–12]. Therefore, *A. niger* has been considered to be the top candidate for the production of other organic acids, e.g., lactic acid, itaconic acid, and malic acid [13–15]. However, succinic acid production by *A. niger* has been explored with little success. Earlier studies on metabolic engineering of *A. niger* have focused on the major metabolic routes that are involved in efficient production of succinic acid by other microorganisms (e.g., reductive tricarboxylic acid (rTCA) branch and glyoxylate bypass). Genetic modifications on these pathways did not lead, however, to an overflow of carbon flux toward succinic acid production [16, 17]. On the other hand, the mechanisms of efficient excretion of organic acids has not been fully understood in *A. niger*, which increases the difficulties in rerouting the carbon flux toward succinic acid from other major organic acids using pathway engineering. In our previous studies, we have identified a key limiting step for C_4_-dicarboxylic acid production in another black aspergillus, *A. carbonarius*, which highly resembles *A. niger* in the organic acid profile [18]. After identifying and overexpressing the native membrane protein AcDCT in *A. carbonarius*, C_4_-dicarboxylic acid production, including malic acid and succinic acid, increased significantly [19]. Another recent study on *A. niger* reported that, efficient production of malic acid was obtained with overexpression of a number of genes related to the cytosolic rTCA branch [15]. These findings imply the high possibility of improving succinic acid production by *A. niger* by continuing carbon flux towards succinate in the cytosolic pathway. Therefore, to test this hypothesis, we chose to express a soluble NADH-dependent fumarate reductase (FRD), which has previously been expressed in a natural malic acid-producing strain of *Aspergillus saccharolyticus* and successfully enhanced production of succinic acid [20], in combination with other genetic modifications in *A. niger*, and evaluated their impacts on succinic acid production (Fig. 1).

Lack of efficient genetic tools has always been a major obstacle to hinder genetic engineering of *A. niger* strains. Although stable ectopic expression of genes in *A. niger* can be achieved with integrative expression vectors, precise genome editing, e.g., gene targeting, remains difficult due to the naturally low homologous recombination frequency in *A. niger* [21]. Moreover, the consecutive genetic manipulations, which are normally required in pathway engineering, are hardly achieved in *A. niger* due to the lack of selectable markers and a time-consuming marker recycling process. Recently, successful applications of the CRISPR–Cas9 system in filamentous fungi have inspired development of novel genome editing tools for *A. niger,* and so far, two types of CRISPR–Cas9 systems have been applied in genetic engineering of *A. niger.* First, an expression-based system using a plasmid vector, which consists of elements for expressing the Cas9 protein and transcription of gRNA in vivo was developed [22]. Then, a ribonucleoprotein (RNP) based system using in vitro assembly of Cas9 protein and synthetic gRNA into a RNP complex, which in turn is applied directly into the protoplast transformation of *A. niger,* was developed [23]. The early-developed expression-based system is restricted to efficient in vivo transcription of gRNA since the stable gRNA products were achieved by expressing a self-cleaved ribozyme-gRNA cassette under regulation of a Pol II promoter [22]. Recently, tRNA promoters have been identified in *A. niger* and applied in transcription of gRNA without ribozyme splicing sequences. However, since the expression of Cas9 protein and gRNA transcription take place in the cell, it is difficult to control the assembly process of Cas9 and gRNA in vivo, which may lead to higher off-target events in the later stage [24]. On the other hand, the RNP-based system adopts the purified Cas9 protein and synthetic gRNA, so it can reach a precise control of gRNA and Cas9 protein in terms of concentrations and provide the optimal reaction conditions for in vitro assembly, which can lower the risk of off-target events. However, although a RNP complex can be used directly in genome editing of *A. niger*, the system naturally lacks a selectable marker for fungal transformation processes. Therefore, the RNP complex is normally used with an additional integrative vector containing marker genes for selection of transformants [25]. In this study, we attempted to use a recyclable marker system together with the RNP complex to realize easy and fast consecutive genetic manipulations in *A. niger*. By evaluating the performance of the engineered strains, we aimed to demonstrate the feasibility of applying *A. niger* as a novel platform for bio-based production of succinic acid from renewable biomass.

## Results

### **Strain engineering **via** RNP-based CRISPR**–**Cas9 system**

In this study, all the genetic modifications were introduced via the CRISPR–Cas9 RNP-based system. The selectable marker was located in the episomal vector pSB-HA containing the AMA1 sequence and transferred separately with RNP complex into fungal cells via protoplast transformation. For single gene disruption, null mutations were introduced into the *gox* and *oah* genes via non-homologous end joining (NHEJ) repair pathway after CRISPR–Cas9-mediated DNA double-strand breaks. The gene disruption efficiency reached 37.5% for the *gox* gene and 12.5% for the *oah* gene. For gene replacement, a donor DNA fragment containing homologous regions (HR) and gene expression cassette were used in addition to the vector pSB-HA and RNP complex in the transformation. As seen in Table 1, two out of 24 transformants were verified for the successful replacement of the *Andct* gene with the *Acdct* gene expression cassette, and two out of 11 transformants had the *frd* expression cassette integrated into the *oah* gene, which yielded efficiencies of 8.3% and 18%, respectively.

**Table 1:** Efficiency of RNP-based CRISPR–Cas9 system for genetic modifications

Genetic modification	Total number of transformants	Number of correct transformants	Efficiency (%)	Corresponding engineered strains
*Δgox*	8	3	37.5	SAP-1
*Δoah*	16	2	12.5	SAP-1
*ΔAnDct::GPDAp-AcDCT-tTrpC*	24	2	8.3	SAP-2
*Δoah::GPDAp-frd-tTrpC*	11	2	18	SAP-3

### Succinic acid production by engineered strains

**Fig. 2:**
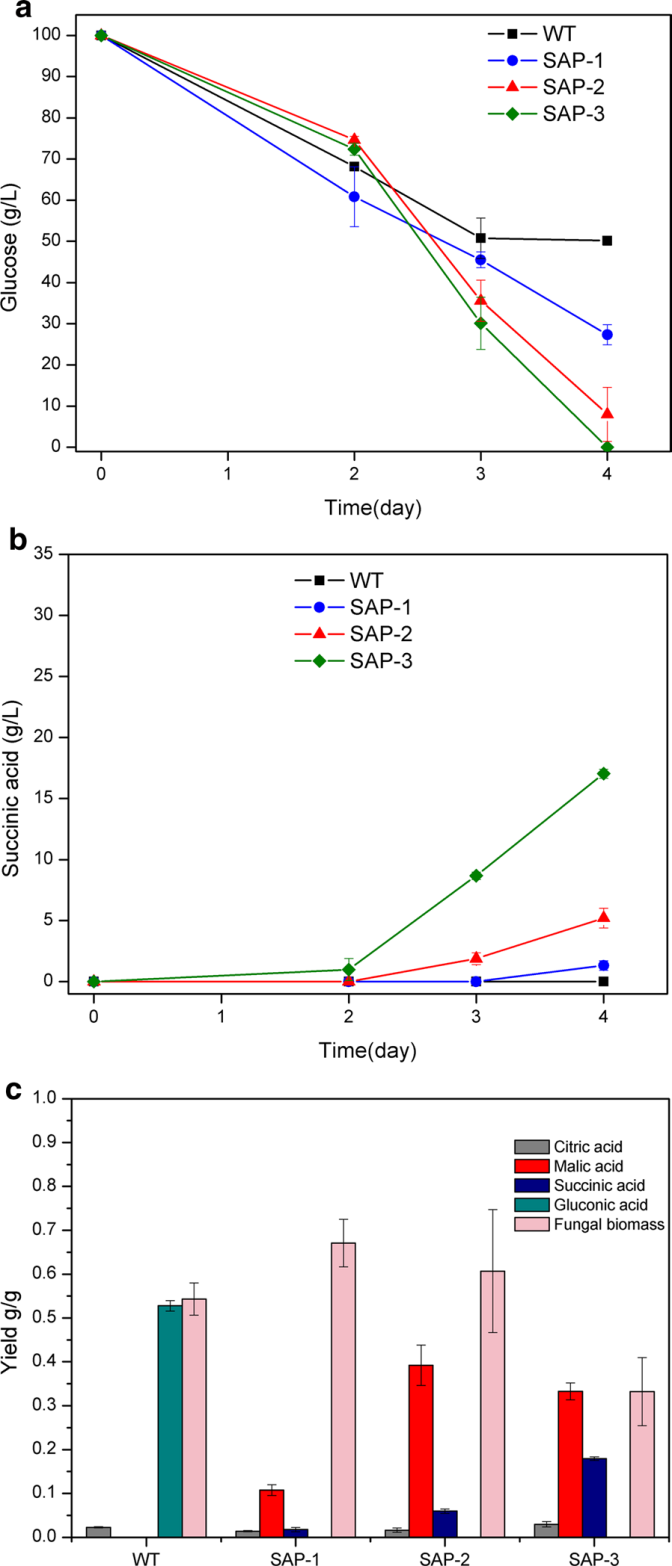
Succinic acid production by the wild type and the engineered strains at 30  °C. **a** Glucose consumption (g/L); **b** production of Succinic acid (g/L); **c** yields of major organic acids and fungal biomass after 4 days

Comparison of succinic acid production by the engineered strains was performed in the defined glucose medium with CaCO_3_ as a pH-buffering reagent. All the strains had pellet morphology during the cultivation (Additional file 1: Table S1). As seen in Fig. 2, the wild type was not capable of producing detectable amount of succinic acid during the cultivation, while most of the glucose was consumed and rapidly converted into gluconic acid (0.53 g/g glucose) after 3 days. In the engineered strains, the SAP-1 strain consumed the lowest amount of glucose after 4 days with significantly lower production of organic acids than the other strains (Fig. 2a, c). However, the SAP-1 strain produced a low amount of succinic acid (0.99 g/L) after 4 days. In the SAP-2 strain, overexpression of *Acdct* gene led to an emergence of succinic acid production in earlier stages of the cultivation and higher amounts of succinic acid production (5.2 g/L) compared with the SAP-1 strain. Furthermore, co-overexpression of the *Acdct* gene and the *frd* gene in the SAP-3 strains resulted in more than threefold increase in succinic acid production than that of the SAP-2 strain, and 17 g/L succinic acid was obtained after 4 days (Fig. 2b). On the other hand, the SAP-3 strain consumed glucose more rapidly than the two other engineered strains, but yielded lower fungal biomass. In an overview of organic acid profiles by the engineered strains, citric acid and malic acid were also produced as major organic acid products in addition to succinic acid. Compared with the wild-type strain, citric acid production was not affected significantly with different genetic modifications, but malic acid production increased in all the engineered strains. However, the yield of malic acid decreased in the SAP-3 strain with overexpression of the *frd* gene compared with the SAP-2 strain (Fig. 2c).

### Impacts of temperature and pH on succinic acid production

**Fig. 3:**
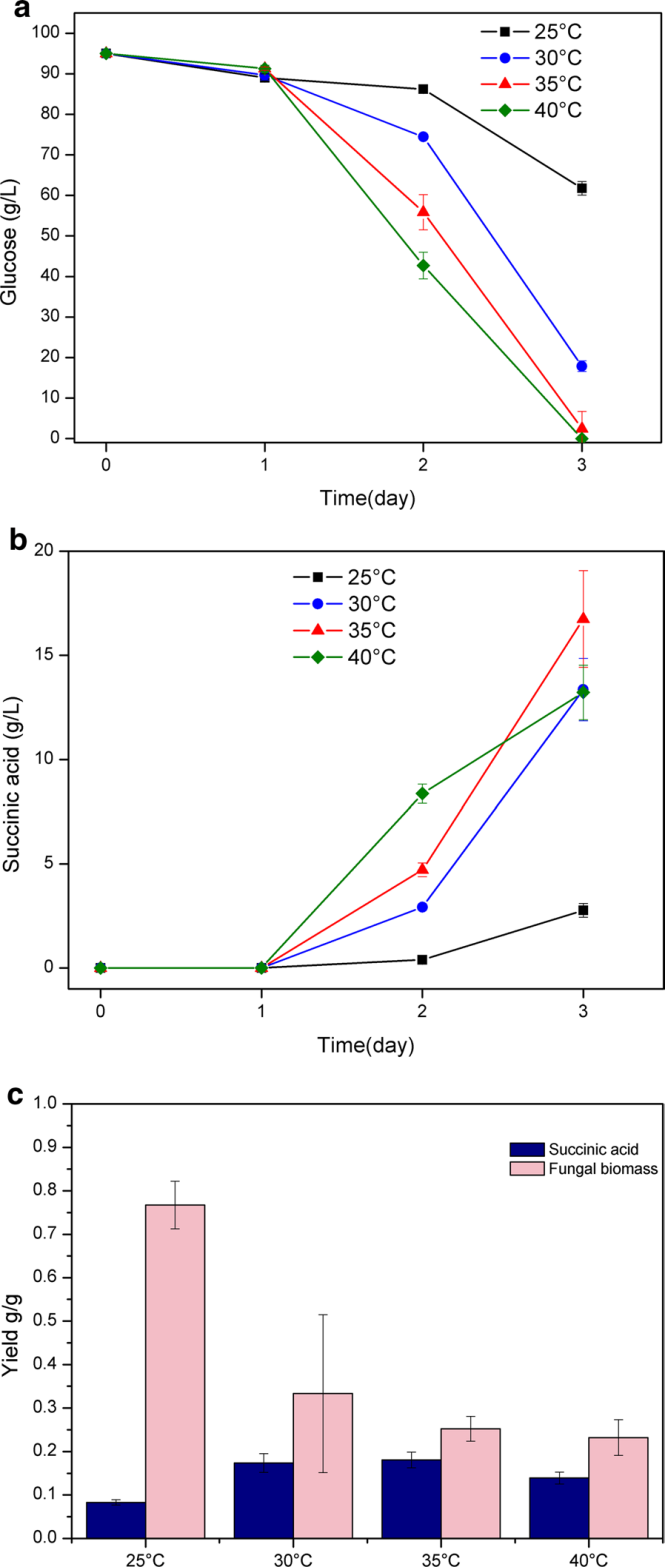
Impacts of cultivation temperature on succinic acid production by the SAP-3 strain. **a** Glucose consumption (g/L); **b** production of succinic acid (g/L); **c** yields of succinic acid and fungal biomass after 3 days

The impact of different temperatures on succinic acid production was investigated by cultivating the SAP-3 strain with the defined glucose medium in shake flasks. Four different temperatures, 25, 30, 35 and 40 °C were chosen within the optimal range for the growth of *A. niger*. All the cultures had a similar pH level on day 3 under different cultivation temperatures (Additional file 1: Table S2). As seen in Fig. 3a, the glucose consumption showed positive correlation with the temperature increase after day 1. The SAP-3 strain consumed all the glucose at 40 °C after 3 days, whereas 62 g/L glucose remained in the culture at 25 °C. In terms of succinic acid production, the highest amount and yield (17 g/L and 0.18 g/g glucose) were obtained at 35 °C on day 3 (Fig. 3b, c). The yield of succinic acid at 30 °C reached 0.17 g/g glucose, which was close to the yield at 35 °C, but the sugar consumption and the amount of succinic acid was lower. In contrast, the highest amount of succinic acid was obtained at 40 °C on day 2 due to the more rapid sugar consumption, but the final yield of succinic acid was lower after 3 days than at 35 °C. On the other hand, the low temperature at 25 °C benefitted the fungal biomass growth over succinic acid production; the biomass yield reached 0.76 g/g glucose, which was around 2- to 3-fold higher than the biomass yield at the other three temperature levels (Fig. 3c).

**Fig. 4:**
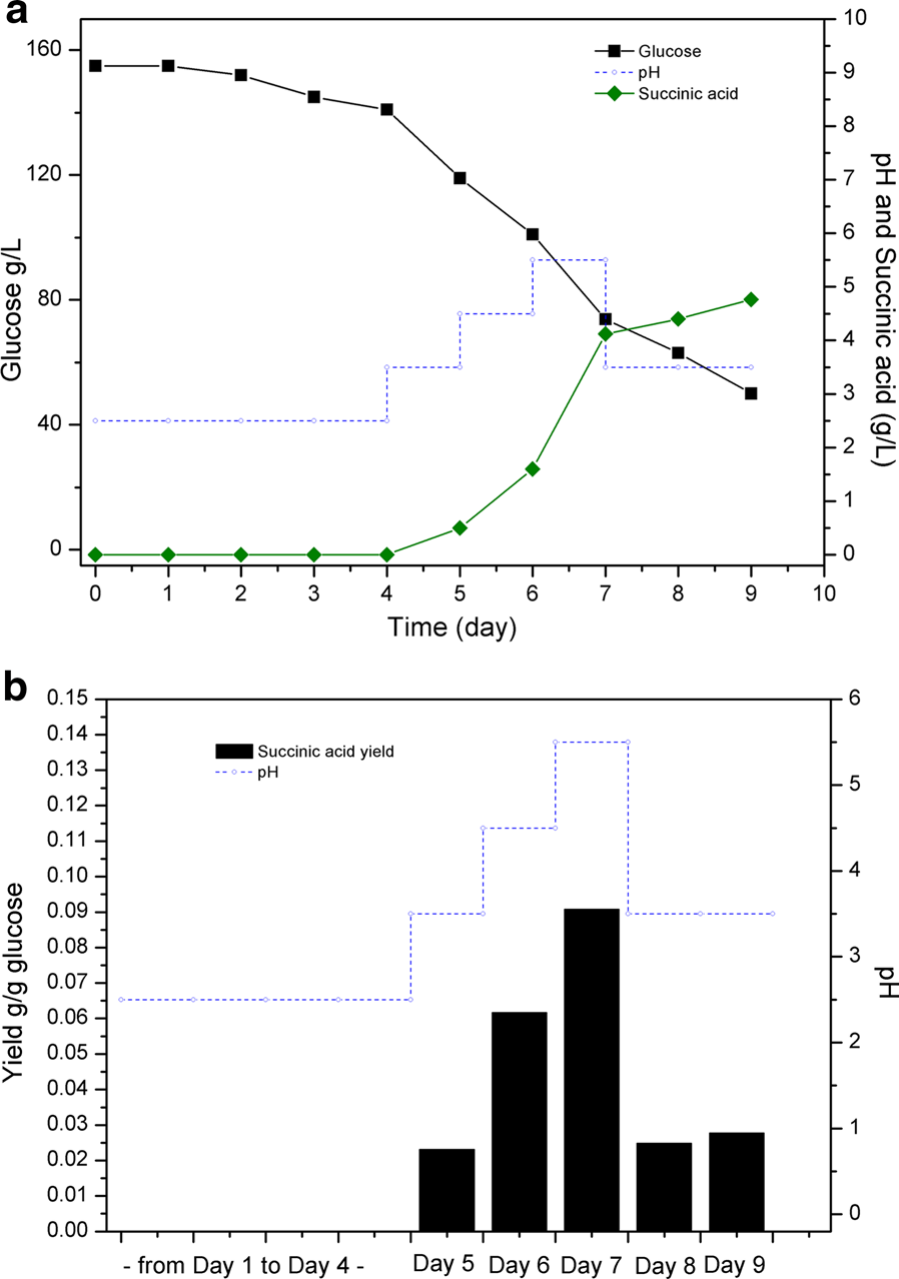
Impacts of low cultivation pH on succinic acid production by the SAP-3 strain at 35 °C. **a** Glucose consumption and succinic acid production (g/L); **b** yield of succinic acid at different pH levels

The impacts of cultivation pH changes on succinic acid production were investigated using a batch culture at controlled pH levels. The cultivation began with a low initial pH at 2.5, and after 4 days, the SAP-3 strain consumed 14 g/L glucose at this pH level, but no measurable amount of succinic acid was detected in the culture. In the following days, the culture pH was adjusted to different levels stepwise and maintained for 1 day. As seen in Fig. 4a, the succinic acid production began after the pH level increased to 3.5 and reached 0.5 g/L with glucose consumption of 22 g/L on day 5. In the next 2 days, the pH levels were adjusted to 4.5 and 5.5, and succinic acid production increased to 1.6 g/L and 4.1 g/L on day 6 and 7, respectively. After day 7, there was still 73 g/L glucose remaining in the culture, the pH level was then lowered again to 3.5. In day 8, the production of succinic acid by the SAP-3 strain only increased to 4.4 g/L (Fig. 4a). Although the sugar consumption was affected due to the pH adjustment in the different stages of cultivation, the yield of succinic acid showed consistently the same trend that higher succinic acid yield was obtained as the pH level was increased. The succinic acid yield reached 0.09 g/g glucose at pH 5.5, and was reduced to 0.03 g/g glucose at pH 3.5 (Fig. 4b).

### Succinic acid production from renewable biomass

**Fig. 5:**
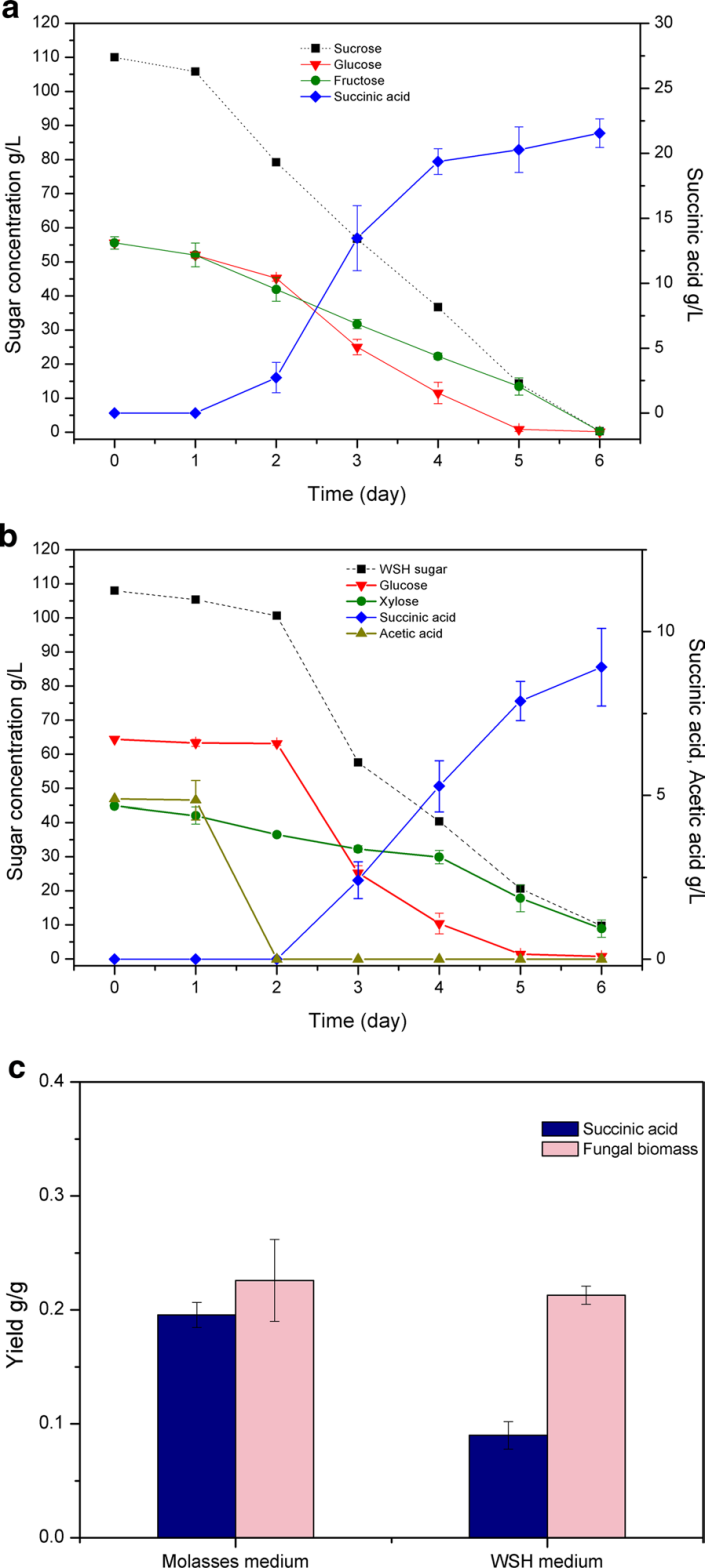
Succinic acid production from sugar cane molasses and wheat straw hydrolysate by the SAP-3 strain 35 °C. **a** succinic acid production and consumption of major sugars in sugar beet molasses; **b** succinic acid production and consumption of major sugars and acetic acid in wheat straw hydrolysate; **c** yields of succinic acids and fungal biomass (WSH—wheat straw hydrolysate)

Two types of renewable biomass, sugar beet molasses and wheat straw hydrolysate were used as substrates for succinic acid production by the SAP-3 strain. Due to the complex compositions of these two substrates, the sugar consumption was monitored by measuring concentrations of major sugars in the substrates: sucrose, which was gradually hydrolyzed into glucose and fructose by *A. niger* in the molasses substrate, and glucose and xylose in the hydrolysate substrate. In molasses, no significant inhibitory effect was observed in the beginning of the cultivation. As seen in Fig. 5a, succinic acid production began after a short lag phase on day 1. After 6 days, the SAP-3 strain consumed all the sucrose and produced 23 g/L succinic acid. On the other hand, as sucrose was gradually hydrolyzed during the cultivation, it was observed that glucose and fructose were consumed at different rates. After 2 days, glucose was consumed more rapidly than fructose with a sharp increase of succinic acid production. In wheat straw hydrolysate, a longer lag phase was observed compared with the molasses medium. As seen in Fig. 5b, measurement of one potential inhibitor in the wheat straw hydrolysate, acetic acid, showed that the SAP-3 strain converted acetic acid prior to the sugar consumption in the first two days. The succinic acid production began from day 3 and reached 9 g/L after 6 days. For sugar consumption, the SAP-3 strain was not able to utilize xylose as efficiently as glucose, where glucose was quickly depleted after day 5, whereas, there was still 9 g/L xylose remaining in the culture after 6 days. Due to the slow xylose consumption, succinic acid production also decreased after day 5. However, even though the succinic acid yield from total consumption of major sugars was significantly lower in the wheat straw hydrolysate than that in molasses, the fungal biomass yields from both media were obtained at similar levels (Fig. 5c).

## Discussion

In this study, we performed consecutive genetic modifications in *A. niger* via the RNP-based CRISPR–Cas9 system in combination with an AMA plasmid containing a dominating selectable marker. The single gene disruptions were achieved using only RNP complexes without any donor HR fragment, where gene disruption efficiencies of 12.5–38% were obtained, which was lower than other CRISPR–Cas9 systems applied in *Aspergillus,* e.g., the plasmid-based system [22, 23]. In vitro assembly of the RNP complex could simplify the construction of the CRISPR–Cas9 system and reduce unspecific off-target cleavage activity of Cas9 with the precise control of the Cas9 protein and gRNA in the assembly process [26]. However, a potential limiting step in the RNP-based CRISPR–Cas9 system is the co-transformation efficiency. Since the uptake of RNP complex in our approach is not subjected to selection pressure, the in vivo concentration of RNP complex is mainly due to the competence of protoplasts and efficiency of co-transformation with the pSB-HA plasmid. Although higher molar concentrations of the RNP was used compared to the plasmid pSB-HA in the protoplast transformation, the actual amount of the RNP complex taken up by the protoplast could not be measured, which increased the uncertainties in the following process of genome editing. On the other hand, the gene replacements were also achieved via co-transformation with RNP and donor DNA containing expression cassettes. In the case of the *oah* gene disruption and replacement with the *frd* gene expression cassette, similar efficiencies were obtained (12.5% and 18%). In this study, the gene disruption and gene replacement were carried out via different DNA repair pathways. After the DNA double-strand breaks were created by the RNP complexes, null mutations were introduced into genes through the DNA repair process in *A. niger*, which highly relies on the mutation frequency of the NHEJ pathway. The gene replacement was achieved through integration of the HR fragments into the genome via the homologous recombination pathway, the efficiency of which is naturally very low in *A. niger* [21]. Therefore, the promising gene replacement efficiencies obtained in this study showed the potential of using an additional knockout cassette for single gene disruption, in this case, the mutations in the target genes can be manipulated more precisely. To further improve the efficiency of genome editing via the RNP-based CRISPR–Cas9 system, the future efforts might be to optimize transformation systems for delivering the RNP complex together with the donor DNA fragment in combination with inactivation of NHEJ pathway, such as using a Ku-deficient strain [24].

As a robust industrial workhorse for citric acid production, *A. niger* has often been used as a good microbial platform for production of other organic acids [12]. The wild-type strain of *A. niger* is capable of accumulating high amounts of citric acid, oxalic acid and gluconic acid from glucose. Especially the latter two organic acids are produced efficiently under the similar conditions that favor succinic acid production, e.g., high glucose concentration and controlled ambient pH levels (from 4 to 6) [27–29]. The two genes, *oah* and *gox*, which are directly involved in production of oxalic acid and gluconic acid [30, 31], were disrupted in the strain SAP-1. The elimination of these two organic acids led to a low production of succinic acid by the SAP-1 strain, which implies that part of the carbon flux has been rerouted towards succinic acid but other limiting steps existing in the relevant pathways still restrict an efficient production. In our previous study, we revealed an important role of the C_4_-dicarboxylic acid transporter for succinic acid production in *A. carbonarius*, which resembles *A. niger* in many features in terms of organic acid production [19]. A recent study on C_4_-dicarboxylate transporters from different fungal species has shown the unique characteristic of AcDCT in relation to its high efficiency of exporting C_4_-dicarboxylic acids [32]. Therefore, we heterologously overexpressed the C_4_-dicarboxylic acid transporter identified from *A. carbonarius* and replaced the original dicarboxylic acid transporter of *A. niger*, which has also been reported for low export efficiency of dicarboxylic acids [15]. The further increase in succinic acid yield in strain SAP-2 compared with the SAP-1 strain indicates that the export of succinic acid is also a limiting step in efficient excretion of succinic acid in *A. niger*. Furthermore, when we overexpressed both the *Acdct* gene and the *frd* gene in strain SAP-3, a synergistic effect of these two genetic modification led to the highest succinic acid production. The soluble NADH-dependent fumarate reductase was expressed to continue the carbon flux towards succinic acid via reduction of the cytosolic precursor fumarate [33], which can normally be produced via the dehydration of malate by fumarase in the rTCA branch [34]. Although fumaric acid was not found at detectable amounts in this study, malic acid was produced by all the engineered strains. Compared with the SAP-2 strain, the SAP-3 strain produced lower amounts of malic acid but more succinic acid, which indicates that part of the carbon flux has successfully been shunted into succinic acid from malic acid. On the other hand, the lowest biomass yield in the SAP-3 strain also implies that the overexpression of FRD also affects the carbon flux between cytosolic reductive pathway and central metabolic pathways in relation with fungal biomass growth, and more carbon flux was rerouted towards organic acid production in the SAP-3 strain.

Moreover, two major cultivation parameters, pH and temperature, were also investigated for their impacts on succinic acid production. *A. niger* has favorable growth temperatures ranging from 25 to 40 °C, and for citric acid production by *A. niger*, the optimal temperature is normally set to 25 to 30 °C [35]. However, our results have shown that the succinic acid production by *A. niger* favors relatively high cultivation temperatures as the highest succinic acid yield was obtained at 35 °C, while the lowest cultivation temperature 25 °C decreased succinic acid production dramatically. Oppositely, the measurement of fungal biomass at different temperatures showed that the carbon flow was increased towards fungal growth instead of succinic acid production as the highest biomass yield was obtained with the lowest sugar consumption at 25 °C. In the case of citric acid production, the higher temperature may cause denaturation of the key enzyme in the TCA cycle, citrate synthase, which in turn hinders the biosynthesis of citric acid [36]. Therefore, a possible explanation for low succinic acid production at low temperature is that the enzymes involved in the cytosolic reductive TCA branch, e.g., malate dehydrogenase and fumarate reductase, which normally have high optimum temperatures, exhibit lower activity at 25 °C [37–39]. The impact of culture pH on succinic acid production was also investigated in the batch culture where the initial pH was set to 2.5. As *A. niger* was able to grow in a broad range of pH values with steady intracellular pH level [40], low cultivation pH was commonly used for citric acid production to avoid accumulation of byproducts [9]. Fungal growth and sugar consumption were observed at pH 2.5, and similar pellet morphology was obtained compared with the shake flask culture (Additional file 1: Table S1), but succinic acid production was not detected in measurable quantity. While the pH level was adjusted stepwise from 3.5 to 5.5, succinic acid production increased gradually as seen in Fig. 4c. To further validate the impact of culture pH changes on succinic acid, the cultivation at pH 3.5 was performed in the batch culture, on day 8. Similar yields of succinic acid obtained on day 5 and 8 at pH 3.5 showed that the succinic acid production has an instantaneous response to pH changes and was suppressed by low culture pH value, especially taking into consideration the fact that the highest succinic acid yield was obtained on day 7, and a high quantity of glucose still remained in the culture. Since *A. niger* strains are not able to naturally produce high amounts of succinic acid, the impacts of pH on succinic acid production have not been examined before. Our results showed that the natural tolerance of *A. niger* to low pH levels did not lead to efficient succinic acid production as known for citric acid production. Compared with the reported succinic acid producers, e.g., *A. succinogenes* and *E. coli*, which are capable of producing 105 g/L and 101 g/L succinic acid from glucose medium [41], one of the major advantages for developing *A. niger* into a new fungal cell factory is to couple its excellent low pH tolerance with low pH process for succinic acid production. As it has not been observed in our engineered strains, the future challenge in strain development of *A. niger* is to identify the mechanism for low pH suppression and improve the efficiency of succinic acid production at low pH levels. The succinic acid production in *A. niger* has similar responses to ambient pH changes as oxalic acid production, which is normally obtained at relatively high pH levels (from pH 3 to 6). Although the mechanism of pH regulation on oxalic acid production in *A. niger* is not fully understood, it has been reported that the expression levels of different genes varies at different pH levels via the regulation of pH dependent transcription factors OafA and PacC [42, 43]. Therefore, further studies on the regulation of genes related to succinic acid-producing pathways may offer the path to efficient production of succinic acid at low pH level.

Sugar as substrate is expensive and it is of interest to find cheap sugar containing substrates for future industrial production. Therefore, two types of renewable biomasses were investigated as substrates for succinic acid production by *A. niger*; sugar beet molasses, which has been used as an industrial feedstock for citric acid production by *A. niger* for decades, and wheat straw hydrolysate, which is obtained from agro-industrial lignocellulosic waste after pretreatment and enzymatic hydrolysis. One of the major challenges to apply renewable biomasses in biological processes is the inhibitory effects on microbial growth due to the chemical composition of the biomass, especially after harsh pre-treatments of lignocellulosic biomass. Compared with the defined medium, a significant inhibitory effect was observed only in the wheat straw hydrolysate but not in the culture containing molasses. Our previous study has shown that a number of inhibitors, especially acetic acid, in the hydrolysate can suppress the spore germination and mycelial growth in the early stage of the fungal cultivation [44]. Normally these inhibitors are converted in the lag phase before sugar consumption begin, which is consistent with the phenomenon that the acetic acid consumption preceded sugar consumption in the wheat straw hydrolysate in this study. In molasses medium, as *A. niger* naturally can resist the potential inhibitors in molasses and efficiently use molasses sugar for organic acid production, the SAP-3 strain produced similar amount of succinic acid from molasses compared with the glucose defined medium. On the other hand, the low succinic acid production from wheat straw hydrolysate indicates the challenges in the utilization of this type of renewable biomass due to their complex composition and the pre-treatment effects. Although similar amounts of sugar were used in the media containing molasses and the wheat straw hydrolysate, the carbohydrate compositions were still different. In molasses, sucrose exists as the major sugar component, which is normally hydrolyzed into glucose and fructose and utilized by *A. niger* as carbon sources. In the wheat straw hydrolysate, the two major types of sugars are glucose and xylose. The consumption of these C6 and C5 sugars shows that glucose was utilized more efficiently than fructose and xylose for succinic acid production. When glucose was present at high concentration in the media, the SAP-3 strain was capable of producing high amounts of succinic acid, but the succinic acid production slowed down when the glucose was depleted. This implies the importance of improving the efficiency of succinic acid production from different types of sugars present in renewable biomass for future pathway engineering of *A. niger*.

## Conclusions

In this study, we performed consecutive genetic manipulations with the RNP-based CRISPR–Cas9 system in *A. niger* ATCC 1015 and improved succinic acid production through pathway engineering. The cultivation temperature and ambient pH value were studied for their effects on succinic acid production, and it is shown that the relatively high temperature (35 °C) and pH level (5.5) in the cultivation benefitted succinic acid production. Furthermore, two types of renewable biomasses, sugar beet molasses and wheat straw hydrolysate, were investigated for their potential to be substrates for succinic acid production by *A. niger*. Since succinic acid production was obtained from both types of substrates at different yields, the feasibility of using renewable biomass for bio-based production of succinic acid by *A. niger* is proven.

## Methods

### Strains and growth conditions

The *A. niger* strain ATCC1015 and all the engineered strains (Table 2) were cultivated on potato dextrose agar plates at 30 °C for spore production. For genomic DNA extraction and preparation of protoplast, all the strains were cultivated in yeast extract peptone dextrose medium (glucose, 20 g/L; yeast extract, 10 g/L; peptone, 20 g/L) at 30 °C overnight.

**Table 2:** List of the engineered strains constructed in the study

Strain name	Description
*∆gox* strain^a^	∆*gox*
SAP-1	∆*gox,* ∆*oah*
SAP-2	∆*gox,* ∆*oah,* ∆*Andct::gpdA*p-*Acdct-trpCt*
SAP-3	∆*gox,* ∆*oah::gpdA*p-*frd-trpCt,* ∆*Andct:: gpdA*p-*Acdct-trpCt*

^a^The *∆gox* strain was constructed and used only as the parental strain for deriving strains SAP-1, 2 and 3 in this study

### Plasmid construction

The Cas9 and gRNA expression cassettes in the plasmid pFC332 (constructed and donated by Technical University of Denmark) were removed by restriction enzymes *Bgl*II and *Bam*HI, and the remaining backbone was self-ligated to form plasmid pSB-HA, which contains the AMA1 sequences together with the *hph* resistance gene (Additional file 1: Figure S1).

The glucose oxidase encoding gene *gox,* oxaloacetate hydrolase encoding gene *oah* and the dicarboxylate transporter encoding gene *Andct* were identified in the JGI genome database *A. niger* ATCC 1015 v4 (Additional file 1: Table S3). For insertion of the upstream and downstream homologous regions of genes (~ 500 bp for each region), *oah and Andct,* individually into expression vectors, two DNA fragments were amplified via PCR from the wild-type genomic DNA with primers OahFw1-OahRv1 and AnDctFw1-AnDctRv1, respectively (Additional file 1: Table S4). The DNA fragments were then cloned individually into the pJET1.2/blunt cloning vector according to manufacturer’s protocol (ThermoFisher®). The resulting HR-containing plasmids were linearized via reverse PCR with primer AnDctFw2/AnDctRv2 and OahFw2/OahRv2 for the following cloning steps. For overexpression of gene *Acdct* (Genbank accession no. KY178298) and *frd* (Genbank accession no. KT026107) in *A. niger*, the AcDCT and FRD expression cassettes were amplified via PCR from a previously constructed plasmid pSBe3Dct and pSBe1Frd, respectively, with corresponding primers. The two expression cassettes were then cloned to corresponding HR-containing plasmids using NEB Gibson assembly kit according the manufacturer’s protocol. All the resulting vectors were verified by DNA sequencing.

### RNP assembly and protoplast transformation

All the crRNA sequences were designed using IDT® online CRISPR–Cas9 guide RNA design tool according to the gene sequences identified in the JGI genome database *A. niger* ATCC 1015 v4 (Additional file 1: Table S3). Cas9 proteins, crRNA and tracrRNA were synthesized and delivered by IDT and the in vitro RNP assembly was carried out as previously described [25]. The final concentration of RNP complex in the assembly mix was 15 µM.

The preparation of protoplasts was carried out as previously described [45]. 100 µL protoplast suspension with concentration of 2 × 10^7^ protoplasts/mL was aliquoted in sterile Eppendorf tubes and used in protoplast transformation. For gene disruption, around 2.5 µg plasmid pSB-HA and 5 µL assembled RNP complex (within a total volume of 10 µL) were added in each transformation reaction. Prior to transformation, the *dct* overexpression cassette was amplified from the corresponding overexpression vector using primers AnDctFw1 and AnDctRv1. For gene overexpression, 2.5 µg plasmid pSB-HA, around 7 µg overexpression cassette/vectors and 5 µL assembled RNP complex (in a total volume of 10 µL) were added to each transformation reaction. All the components were gently mixed in an Eppendorf tube and placed on ice for 15 min. Next, the transformation mixture was incubated at room temperature for 15 min after which 1 mL 40% PEG4000 solution was added into the tube. The following procedure of transformation was performed as previously described [45]. All the transformation plates contained 200 µg/mL hygromycin for selection of transformants. After 3–4 days, the transformants were transferred individually to PDA plates and grown at 30 °C for the screening test.

### Validation of genetic modifications and removal of selectable marker

For gene disruption, the genome regions containing the RNP recognition sites were amplified via PCR using Hi-Fi Q5 polymerase (NEB®) with corresponding primers (Additional file 1: Table S4), the obtained PCR amplicons were purified and sequenced to screen mutations in the genes. For gene overexpression, integration of the expression cassette in target regions of the genome were verified by amplifying the fragments with expected sizes in PCR with *Taq* polymerase. For removal of selectable marker, the transformants were overgrown in PDA plates without hygromycin for 4–5 days and transferred to a new PDA plate by streaking with low amounts of spores. After overnight cultivation at 30 °C, single colonies were isolated and transferred to new PDA plates again for cultivation. After sporulation, the spores were harvested and inoculated to PDA plates containing 200 µg/mL hygromycin. The isolated colonies, which were not able to grow in hygromycin-containing plates, were selected and preserved for the further experiments.

### Succinic acid production

For comparing succinic acid production between the wild type and the selected transformants, the cultivation was carried out in 250-mL Erlenmeyer flasks containing 50 mL succinic acid production medium at 30 °C with agitation speed of 200 rpm. The SAP medium consisted of glucose, 100 g/L; NH_4_NO_3_, 2.5 g/L; KH_2_PO_4_, 0.5 g/L; MgSO_4_ 7H_2_O, 0.6 g/L; CaCl_2_ 2H_2_O, 0.05 g/L; ZnSO_4_, 0.0015 g/L; FeSO_4_ 7H_2_O, 0.1 g/L; biotin, 2 × 10^–5^ g/L and CaCO_3_, 80 g/L. Calcium carbonate was used as a pH-buffering reagent in shake flasks to neutralize the organic acids produced during the cultivation as previously described [15, 19]. For studying the impact of cultivation temperature on succinic acid production, the cultivation was carried out in the same set-up as described above at temperatures set at 25, 30, 35 and 40 °C. For succinic acid production from renewable biomasses, sugar beet molasses and wheat straw hydrolysate were filter sterilized and supplemented with NH_4_NO_3_, 0.2 g/L; KH_2_PO_4_, 0.1 g/L; MgSO_4_ 7H_2_O, 0.3 g/L; FeSO_4_ 7H_2_O, 0.05 g/L; biotin, 2 × 10^–5^ g/L and CaCO_3_, 80 g/L. The final concentrations of sugars were 110 g/L sucrose in the molasses medium and 64 g/L glucose and 45 g/L xylose in the wheat straw hydrolysate medium. In all the cultivations in shake flasks, freshly harvested spores were used and inoculated at concentration of 2 × 10^5^ spores/ mL.

Succinic acid production with controlled pH conditions was performed in 1.5 L medium in a 5-L bioreactor. The medium consisted of glucose, 150 g/L, NH_4_NO_3_, 2.5 g/L; KH_2_PO_4_, 0.5 g/L; MgSO_4_ 7H_2_O, 0.6 g/L; CaCl_2_ 2H_2_O, 0.05 g/L; ZnSO_4_, 0.0015 g/L; FeSO_4_ 7H_2_O, 0.1 g/L and biotin, 2 × 10^–5^ g/L. The temperature and agitation speed were set to 35 °C and 600 rpm, and inlet air-flow rate was maintained at 1vvm. The pH was adjusted and maintained by adding 5 M HCl solution and alkaline solution containing 2 M NaCO_3_ and 1 M NaOH. The spores of the transformant SAP-3 were inoculated at final concentration of 2 × 10^5^ spores/ mL.

### HPLC analysis and fungal biomass measurement

For analysis of organic acids, all the samples were acidified with 50% (v/v) sulfuric acid and incubated at 80 °C for 15 min. The acidified samples were centrifuged at 11,000 rpm for 1 min, and the supernatant was filtered with a 0.45-µm filter and used for HPLC analysis. For analysis of sugar, the samples were prepared in the same process as described above without the acidification step. Analysis of organic acids was carried out with an Aminex 87H column (Biorad®) at 60 °C by using a HPLC mobile phase (5 mM H_2_SO_4_) at a flow rate of 0.6 mL/min, and analysis of sugars was carried out with an Aminex 87P column (Biorad®) at 80 °C by using a HPLC mobile phase (ultrapure water) at a flow rate of 0.5 mL/min.

For fungal biomass measurements, 5 mL culture was acidified and washed with 1 N HCl on a pre-dried 0.22 µm filter until the pH in the flow-through was lower than 2. The filtered fungal biomass was then thoroughly washed with distilled water and dried on the filter paper at 100 °C for 48 h before weighing.

## Supplementary Information

**Additional file 1: Table S1.** Strain morphology in succinic acid production. **Table S2.** The initial and final pH value in the cultures at different cultivation temperatures. **Table S3.** Genes manipulated in this study using corresponding gRNAs. **Table S4.** Primers used in this study. **Figure S1.** Plasmids used in this study.

## Data Availability

All the data analyzed during this study have been included in this published article.

## Abbreviations

FRD: Fumarate reductase
RNP: Ribonucleoprotein
SAP: Succinic acid producing
rTCA branch: Reductive tricarboxylic acid branch
