# Supplementary material for: Metabolic engineering of Aspergillus niger via ribonucleoprotein-based CRISPR–Cas9 system for succinic acid production from renewable biomass
Source: Biotechnol Biofuels. 2020 Dec 14;13:206. doi: 10.1186/s13068-020-01850-5 (PMC7737382; doi:10.1186/s13068-020-01850-5)
Supplement: Supplementary file 1 — Additional file 1: Table S1. Strain morphology in succinic acid production. Table S2. The initial and final pH value in the cultures at different cultivation temperatures. Table S3. Genes manipulated in this study using corresponding gRNAs. Table S4. Primers used in this study. Figure S1. Plasmids used in this study. [file 13068_2020_1850_MOESM1_ESM.docx]

**Metabolic engineering of *Aspergillus niger* via Ribonucleoprotein based CRISPR-Cas9 system for succinic acid production from renewable biomass**

**Lei Yang^1^*, Mikkel Møller Henriksen^1^, Rasmus Syrach Hansen^1^, Mette Lübeck^1^, Jesper Vang^1#^, Julie Egelund Andersen^2^, Signe Bille^3^, Peter Stephensen Lübeck^1^**

^1^Section for Sustainable Biotechnology, Department of Chemistry and Bioscience, Aalborg University Copenhagen, A. C. Meyers Vænge 15, DK-2450 Copenhagen SV, Denmark

^2^Section of Microbiology, Department of Biology, University of Copenhagen, Universitetsparken 15, DK-2100 Copenhagen, Denmark

^3^Section of Cell and Neurobiology, Department of Biology, University of Copenhagen, Universitetsparken 15, DK-2100 Copenhagen, Denmark

^#^Present address: Disease Data Intelligence, Department of Health Technology Bioinformatics, Technical University of Denmark, Kemitorvet, Bldg. 208, DK-2800 Kgs. Lyngby Denmark

*Corresponding author: Telephone +45 50289330, Fax +45 99402594, Email [ly@bio.aau.dk](mailto:ly@bio.aau.dk)

**Table S1:** Strain morphology in succinic acid production

| WT strain | SAP-1 strain | SAP-2 strain | SAP-3 strain | SAP-3 strain |
| --- | --- | --- | --- | --- |
| 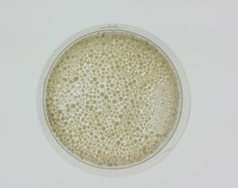 | 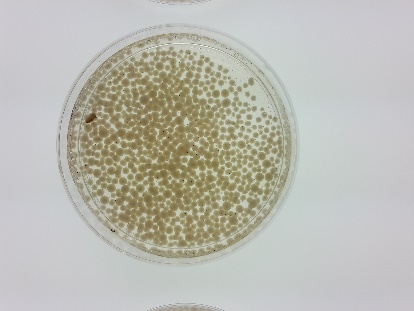 | 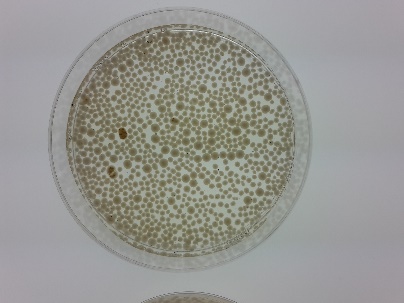 | 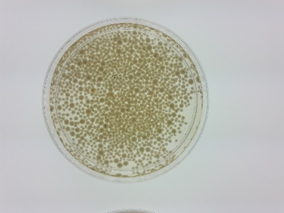 | 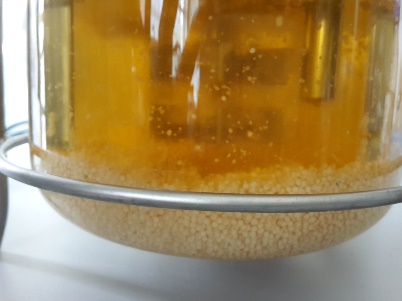 |
| In shake flasks | In shake flasks | In shake flasks | In shake flasks | In bioreactor |

**Table S2:** The initial and final pH value in the cultures at different cultivation temperatures

| pH value | 25 ^o^C | 30 ^o^C | 35 ^o^C | 40 ^o^C |
| --- | --- | --- | --- | --- |
| Initial pH (culture medium pH) | 6.15 | 6.15 | 6.15 | 6.15 |
| Final pH ( on Day 3) | 5.73±0.07 | 5.79±0.02 | 5.74±0.04 | 5.74±0.12 |

**Table S3:** Genes manipulated in this study using corresponding gRNAs

| **Gene** | **Protein ID** | **Location** | **gRNA used (5’🡪3’)** |
| --- | --- | --- | --- |
| *gox* | 1132679 | chr_202: 3673369-3675186 | GTCACCAAAGGTCTCGTTGA |
| *oah* | 1145268 | chr_501: 212890-214547 | GCCCTCGAGGAGACCAACAT |
| *Andct* | 1166397 | chr_503: 414792-416042 | CCGGTGTACAATAGTAGTAC |

**Table S4:** Primers used in this study

| Name | Sequence (5’→3’) | | Annotation |
| --- | --- | --- | --- |
| AnDctFw1 | GGAGAAATCAGCGCACGTTG | PCR amplification of dct homologous regions | |
| AnDctFw2 | TGTGTCAGCCTTTCTACGACGCC | Reverse PCR amplification of *dct* HR-containing plasmids | |
| AnDctRv1 | CGTTCACAGTCCAGTGCCTCCG | PCR amplification of dct homologous regions | |
| AnDctRv2 | AGAATTGATGGTATGCGCCAC | Reverse PCR amplification of *dct* HR-containing plasmids | |
| GpdFw | TCGTTGACCTAGCTGATTCTGGCATACCATCAATTCTTCGTGGACCTAGCTGATTCTGG | Amplification of *Acdct* and *frd* overexpression cassettes with Gibson Assembly overhangs | |
| TrpRv | TCGAGTGGAGATGTGGAGTGGTCGTAGAAAGGCTGACACATCGAGTGGAGATGTGGAGTG | Amplification of *Acdct* and *frd* overexpression cassettes with Gibson Assembly overhangs | |
| OahFw1 | TTGGCTAGGCTACTATCTTC | PCR amplification of *oah* homologous regions | |
| OahFw2 | ATTAAACCCTCAGCGCGGCC CGAAAAATGTCCGAATGCAA | Reverse PCR amplification of *oah* HR-containing plasmids with Gibson Assembly overhangs | |
| OahFw3 | CACCATGGACGAATACTTGAC | Amplification of PCR amplicon for DNA sequencing to validate the *oah* gene disruption | |
| OahRv1 | AGTGGGACCAATAGTAACTG | PCR amplification of *oah* homologous regions | |
| OahRv2 | GAGGTAATCCTTCTTTCTAG TTCCTGCTTCTCACTCCAT | Reverse PCR amplification of *oah* HR-containing plasmids with Gibson Assembly overhangs | |
| OahRv3 | TAATGCAAGCGAACGAGAAG | Amplification of PCR amplicon for DNA sequencing to validate the *oah* gene disruption | |
| GoxFw | CTGTCGAAGACCGGGGCGTT | Amplification of PCR amplicon for DNA sequencing to validate the *gox* gene disruption | |
| GoxRv | GCACACCATACACACGGGCA | Amplification of PCR amplicon for DNA sequencing to validate the *gox* gene disruption | |


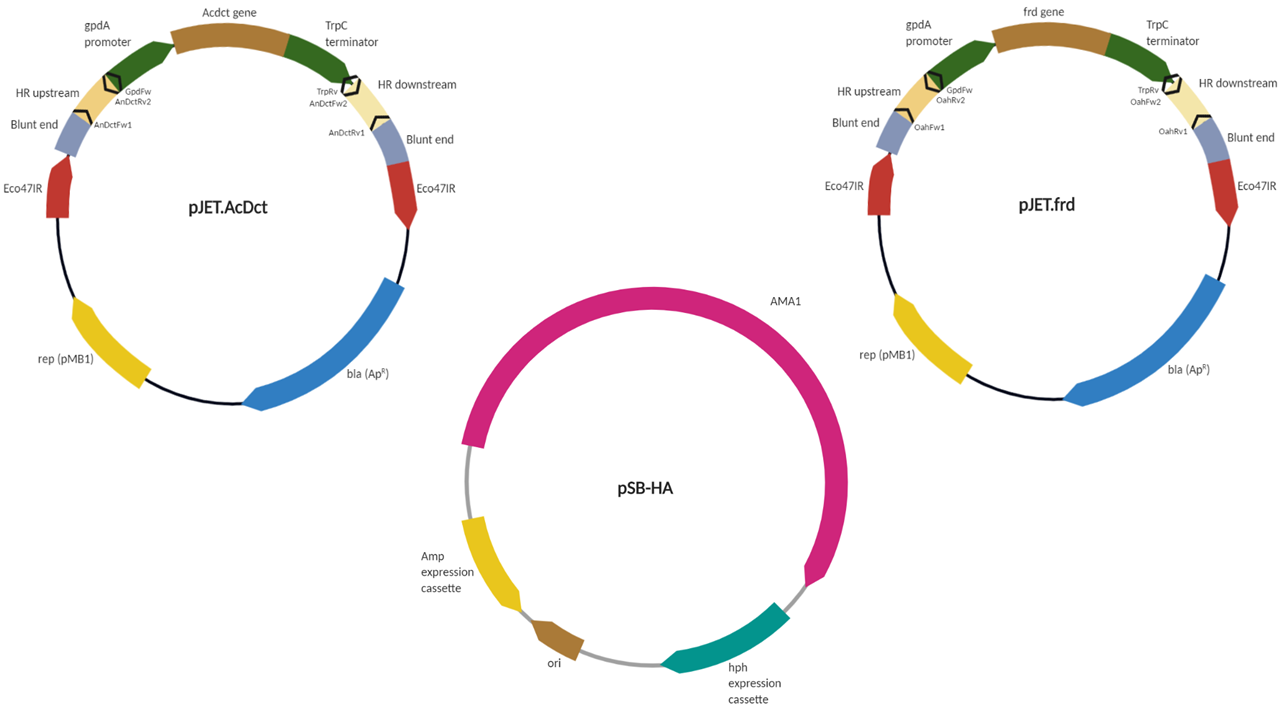


**Figure S1.** Plasmids used in this study. gpdA, constitutive promoter; TrpC, terminator; hph, hygromycin resistance gene; Amp, ampicillin resistance gene; ori, origin of replication in the plasmid; rep, replicon responsible for replicating pJET1.2; bla (Ap^R^), β-lactamase gene providing ampicillin resistance; Eco47IR, lethal gene providing positive selection of recombinant E. coli cells; frd, fumarate reductase encoding gene; Acdct, A. carbonarius dicarboxylate transporter encoding gene.
